# Cellular and molecular basis of Venous insufficiency

**DOI:** 10.1186/s13221-014-0024-5

**Published:** 2014-12-12

**Authors:** Elizabeth S Pocock, Tom Alsaigh, Rafi Mazor, Geert W Schmid-Schönbein

**Affiliations:** Department of Bioengineering, The Institute for Engineering in Medicine, University of California San Diego, 92093-0412 La Jolla, California

**Keywords:** Vein, Venous disease, Molecular, Genetics, Valves, Endothelial, Blood vessel

## Abstract

Chronic venous disease (CVD) has a range of clinical presentations, including tortuous, distended veins in lower extremities, increasing skin pigmentation, and in severe cases ulceration of the affected skin. Venous insufficiency, a precursor to CVD characterized by improper return of blood from the lower extremities to the heart, must be studied in its earliest stages at a time when preventative measures could be applied in man. This underscores the need for basic research into biomarkers and genetic predisposing factors affecting the progression of venous disease. Investigation over the past decade has yielded insight into these specific genetic, cellular and molecular mechanisms underlying the development of venous disease. Among the many advances include the elucidation of an increasing role for matrix metalloproteinases as important mediators of the degenerative process involved with venous insufficiency. This may be preceded by an inflammatory process which further contributes to venular degeneration and endothelial dysfunction seen in advanced presentation of disease. Furthermore, genomic analyses have shed light upon temporal expression patterns of matrix remodeling proteins in diseased tissue samples. In this review we examine some of the current findings surrounding cellular, molecular and genetic advances in delineating the etiology of chronic venous disease.

## Introduction

A variety of mechanisms have been proposed as the etiology of chronic venous insufficiency. One common hallmark of this disease is an elevated venous pressure [1-4]. Valve coaptation failure is an early inciting event; it is associated with venous wall distension, but the underlying reason for this occurrence remains unexplained. Other causes, such as perivascular fibrin cuffs, ischemia-hypoxia, and growth factor trapping, have been proposed, and there is evidence for white blood cell infiltration as a marker for an inflammatory cascade [5]. The inflammatory cascade serves in life largely as a tissue repair mechanism, and therefore this evidence poses the question of what mechanism triggers the inflammation and may sustain it [6-9].

Venous hypertension results in vein wall distension and loss of the normal fluid shear stress [10-12]. These changes in the biomechanical environment of veins facilitate a variety of cellular responses: they alter the transport of inflammatory mediators (for example, accumulation of cytokines), and facilitate multiple other cell dysfunctions. The role of inflammation comes closely into focus to understand the etiology of chronic venous insufficiency.

### Collagen/Elastin

Varicose veins are composed of tortuous and non-tortuous segments [13]. The non-tortuous segments are similar in structure to a healthy vein. These, however, are interspersed with tortuous segments, which display alterations in collagen and elastin content throughout the wall. The tortuous segments have non-uniform structure with alternating regions of hypertrophy and atrophy [13-15]. Atrophied regions have decreased extracellular matrix and smooth muscle cells, while hypertrophied regions have disruption of the smooth muscle cells with an increased amount of extracellular matrix [15]. There is conflicting data regarding collagen content in varicosities, although most studies have demonstrated that it is increased compared with healthy veins [13,14,16,17].

Xiao et al. demonstrated increased collagen synthesis in smooth muscle cell cultures from varicose veins compared with healthy controls [18]. There are multiple subtypes of collagen, which have a variety of roles. Veins are nonlinear viscoelastic structures. Type I collagen tends to contribute the major mechanical stress to the stiff properties of veins at higher levels of distension, while Type III collagen provides also a stress at lower levels of distension [19,20]. Multiple studies have demonstrated an increase in Type I, and decrease in Type III, collagen in similar cell cultures, as well as varicose vein tissue [19-21]. Jeanneret et al. demonstrated that decreased Type III collagen in varicose vein segments, as compared with controls without known venous insufficiency, correlated with increased distensibility with Valsalva [20]. Of note, cell culture from dermal fibroblasts of patients with varicosities demonstrated similar findings, suggesting a systemic alteration in collagen synthesis [19].

Studies focusing on the layers of the vein wall reveal a variety of alterations in chronic venous insufficiency. The intima has a disrupted elastic lamina with alterations in collagen content [20,22,23]. Smooth muscle cells within the lamina are increased both in number and size [15]. Within the media, the smooth muscle cell bundles are disrupted by increased content of collagen and elastin and development of fibrosis, as demonstrated by histology, with increased quantities of hydroxyproline, a collagen precursor [16,19,24]. The disruption of the vein wall structure goes hand in hand with dilatation of the vein wall in varicosities, aided by the lack of contact with smooth muscle cells and impairment of the contractile properties of the vein tissue [16]. The adventitia has decreased elastin [20].

The amount of elastin appears to be inversely related to the diameter of a vessel [20]. With age, there are decreased levels of elastin synthesis, as evidenced by components such as tropoelastin and lysyl oxidase-like 1 (LOXL-1) [25]. However, in patients with varicosities, there is an even greater decrease in LOXL-1 than in age-matched controls [25]. Sansilvestri et al. demonstrated thickened and fragmented elastic fibers not only in varicose vein segments but also in the skin of patients with chronic venous insufficiency [17]. This evidence provides further support for a potential systemic alteration in extracellular matrix in individuals with venous insufficiency.

### Extracellular matrix components/glycoproteins

There are a variety of other alterations in the extracellular matrix in chronic venous insufficiency. Laminin, which plays an important role in remodeling and extracellular matrix organization, is increased in varicose vein tissue [17]. In addition, in both varicose vein tissue and the skin of the patients with varicose vein disease there is an increase in fibrillin-1, a key structural component of elastic fibers [17,26]. A 2005 study found increased transforming growth factor-beta (TGF-β) in varicose vein tissue, compared with control [13]. A significant difference was also noted within the varicose tissue, with higher TGF-β in areas of tortuousity [13]. A more recent study confirmed this finding, and demonstrated an increase in active TGF-β in varicose vein tissue [27]. Interestingly, fibrillin 1 has recently been shown to play a role in the limiting the activation of TGF-β [28].

### Endothelial activation

Endothelial cells are exquisitely sensitive to their mechanical environment, for example, the degree of fluid shear stress on their plasma membrane, the stretch due to vessel distension, and the stiffness of the extracellular matrix to which they are attached. Many of these factors are altered in varicose veins and may thus influence the behavior of endothelial cells. Multiple studies have demonstrated endothelial activation in the veins of patients with varicosities. This serves as a major contribution to the proinflammatory state in chronic venous insufficiency. For example, exposing endothelium to increased pressure results in enhanced synthesis of VCAM, ICAM, and ELAM. In patients with varicose veins, ICAM, VCAM, laminin, E-selectin, and ELAM are elevated in the tissue and in serum in a soluble form [9,29-31].

### Endothelial dysfunction

Carrasco et al. demonstrated impaired endothelial-dependent relaxation in response to acetylcholine in varicose vein segments [32] just as in diseased arteries. In addition, unlike previous studies that demonstrated decreased contractility to noradrenaline stimulation, this study also found an increased response [32]. The authors proposed that this may be a compensatory response to prolonged distension of varicose veins.

### Matrix metalloproteinases

Matrix metalloproteinases (MMPs) are involved in extracellular matrix degradation and may thus play a central role in the restructuring of veins in chronic venous insufficiency [14]. Multiple studies have confirmed increased levels of tissue and plasma MMPs in chronic venous insufficiency, including MMP-1, MMP-2, MMP-3, MMP-9, and MMP-13 [17,18,33-35].

Gillespie et al. demonstrated an MMP-1 increase in varicose vein segments, although to a lesser extent at the distal aspect of the varicosity [33]. The same study demonstrated significantly less MMP-13 protein levels at the distal extent of a varicosity, as compared with the proximal aspect [33].

MMP-2 and MMP-3 degrade elastin and collagen, abnormalities frequently identified in venous disease [17,18]. Smooth muscle cells cultured from varicose veins have increased MMP-2 compared with healthy controls [18]. Sansilvestri-Morel et al. demonstrated increased MMP-1, MMP-2, and MMP-3 in both varicose vein tissue and the skin of patients affected with chronic venous disease [17].

Raffetto et al. demonstrated in an animal model relaxation of the inferior vena cava in response to MMP-2 [35]. These investigators propose that this relaxation could be related to smooth muscle cell hyperpolarization secondary to a calcium-dependent potassium channel BK_Ca_ [35]. In a follow-up study of rat chronic venous insufficiency segments exposed to increased tension, the same group demonstrated increased MMP-2 and MMP-9, and reduced contractility of the vein segment [34]. They propose this as a potential mechanism for the venous dilatation seen in chronic venous disease.

There is a conflicting record regarding MMP-2, with several studies demonstrating decreased levels of this enzyme in varicose vein tissue [14,36]. This could result in extracellular matrix accumulation, and in possibly contribution to the alternating regions of hypertrophy and atrophy in varicosities [14]. In addition, both groups that identified decreased levels of MMP-2 also demonstrated increased levels of tissue inhibitors of metalloproteinese (TIMP-1).

**Figure 1:**
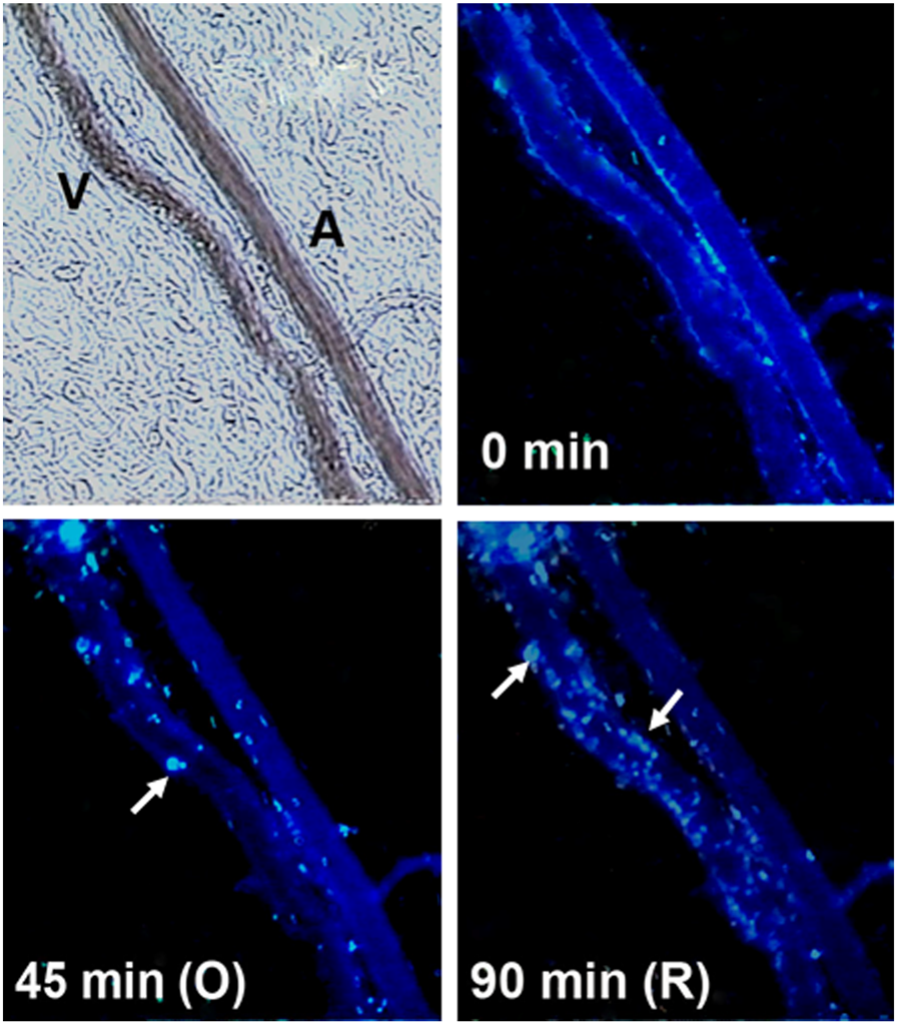
**Micrograph of post-capillary venule upstream of occlusion point.** Arrows point to the release of active MMP-8 from accumulating adherent and rolling leukocytes (leukocyte-fluorescence co-localization). V = venule; A = arteriole. Length bar = 100 μm.

There have been recent efforts to detail the early versus late MMP expression patterns in venous disease. Alsaigh et al. examined MMP activity *in vivo* over an acute 90-minute occlusion and reperfusion of postcapillary venules in the rodent mesentery. Utilizing microzymographic techniques, this group determined that accompanying a short rise in local blood pressure together with a reduction of the fluid shear stress, there is early expression of active MMP-1, MMP-8, and MMP-9, with MMP-1 and MMP-9 being released from endothelial cells and MMP-8 being released from rolling and adherent leukocytes (Figure 1). There was also an increased presence of TIMP-1 and TIMP-2, again suggesting an important interplay between MMPs and their inhibitors in the early stages of venous occlusion. The relatively early expression of MMPs (within minutes) detected in this study suggests that the degradative activity of these proteases may be early culprits that initiate the inflammatory reactions responsible for the breakdown and restructuring of the venous wall seen in venous disease [37].

Depending on the particular MMP involved, the MMP activity may not only be associated with breakdown of extracellular matrix proteins but also cleavage of membrane receptors, such as vascular endothelial growth factor-2 (VEGFR-2) [37]. Such receptor cleavage may compromise endothelial viability.

### Cytokines

Typical for all inflammatory states, a shift in cytokine expression pattern is encountered in varicose vein tissue. These cytokines serve for the most part the repair side of inflammation and are synthesized by intact cells that involve endothelium, macrophages, smooth muscle cells, and others. TGF-β_1_ has a variety of roles, including a key role in vascular remodeling [27]. It recruits macrophages and fibroblasts and inhibits expression of MMP-1 [10,38]. In addition, in the presence of other growth factors, TGF-β_1_ inhibits collagenase synthesis and synergistically increases TIMP expression [39]. TGF-β_1_ is increased in varicose veins compared with normal tissue [13,27]. This difference has been further broken down to demonstrate increased TGF-β_1_ in the tortuous segments of varicose veins, as compared with non-tortuous segments [13].

Isoform nitric oxide synthase iNOS is present only at low levels in normal non-injured tissue. However, in varicose vein tissue, it is significantly elevated [13]. Similar to the distribution pattern of TGF-β_1_, iNOS is present in greater amounts at the tortuous segments of varicosities, compared with non-tortuous segments [13].

In addition, Shoab et al. demonstrated increased vascular endothelial growth factor (VEGF) in patients with chronic venous disease [40].

### Leukocytes

Already during early stages of inflammation, leukocytes roll along the endothelium utilizing the cellular adhesion molecule L-selectin. Once stimulated, leukocytes shed this molecule and express integrins to facilitate extravasation into the tissue [12]. A variety of studies have demonstrated increased degranulation and extravasation of leukocytes in patients with venous hypertension.

Saharay et al. demonstrated decreased neutrophil and monocyte L-selectin as detected with flow cytometry in patients with chronic venous insufficiency with venous hypertension [41]. Interestingly, in a follow-up study in 2000 by Junger et al., no shift in expression of L-selectin on neutrophils or monocytes was demonstrated in healthy controls compared with patients with chronic venous insufficiency following experimental venous hypertension [42]. The study by Junger demonstrated a decreased expression of L-selectin in lymphocytes, however [42].

### Lymphocytes

T-lymphocytes have also been shown to be at least partially involved in the inflammatory process seen in venous disease. By analyzing blood obtained from varicose veins of patients with CVD, Ojdana et al. were able to show that levels of CD4+ T lymphocytes measured from varicosities was significantly elevated compared to overall measured levels of CD4+ cells. The increased level of these cells, as well as other T-cell subpopulations measured by this group, indicate that T-cells may also be partially involved in the pathogenesis of CVD [43].

### Mast cells

Mast cells can carry out a wide variety of functions that mediate the inflammatory cascade by mediators derived from their granules on endocytosis. Pascual et al. demonstrated a significantly elevated infiltration of mast cells into varicose vein walls, as opposed to healthy controls [27]. Mast cell chymase is an activator of MMP-1 (a procollagenase) and MMP-3 [27,44], and also stimulates the release of TGF-β, which plays an integral role in vascular remodeling [27]. Mast cells also secrete tryptase, which can degrade elastin, Type IV collagen, proteoglycans, and fibronectin [27].

### Smooth muscle cells

Smooth muscle cells contribute distinctly to many aspects of the elasticity of a vein. Varicose veins have decreased layers of smooth muscle cells in the subintima of the wall, as well as a disrupted and disorganized smooth muscle structure within the media [13,24]. But the decreased quantity of smooth muscle cells is accompanied by an increased cell size [13]. An interesting study by Knappen et al. supports the finding of increased smooth muscle cell volume, correlated with an increased estrogen receptor-β expression [45].

The smooth muscle cells within varicosities have demonstrated altered function, as well as size. Metcalfe et al. found that smooth muscle cells of varicose vein have decreased contractility compared with controls [15]. This was linked to a decrease in the purinergic P2X_1_ receptor, an adenosine triphosphate-gated cation channel [15]. The investigators demonstrated a transition from contractile to synthetic function of smooth muscle cells, as evidenced by increased intracellular organelles, as well as upregulated purinergic receptor subtypes P2Y_1_ and P2Y_2_ [15]. A subsequent study by Xiao et al. demonstrated significantly decreased levels of alpha-smooth muscle actin, smooth muscle myosin heavy chain, and smoothelin, components integral for the contractile function of the smooth muscle cell [18].

### Valves

Valve destruction and valve coaptation failure are key contributors to venous insufficiency and hypertension [46]. Angioscopic evaluation shows a decreased number of valves in the great saphenous vein in patients with chronic venous insufficiency [46].

In addition to a decrease in valve number in chronic venous insufficiency, the structure of the valve is affected as well. Increased pressure at the valve, as well as decreased shear stress, can trigger an inflammatory cascade, with subsequent valve damage and remodeling [47]. A study of patients undergoing surgery for chronic venous insufficiency found infiltration of valve leaflets and the vein wall by monocytes and macrophages [48]. These tissues also demonstrated destruction of collagen and disruption of the elastic lamina, with redistribution of elastic fibers throughout the vein wall.

### Lymphatics

Recent studies have also examined the specific genes involved in venous valve morphogenesis [49]. A report by Bazigou et al. makes the important discovery that developing venous valves expressed, among other proteins, prospero-related homeobox1 (Prox1), VEGFR3, and integrin-a9, all previously characterized as important regulators of lymphangiogenesis [50,51]. This group further suggests that the development of these two cell types (venous endothelial and lymphatic endothelial) may display plasticity upon normal development; the molecular switches controlling lymphatic development may also control venous valve development, and these switches may be controlled, in turn, by contextual environmental cues such as blood flow and shear stress. These observations shine a bright light on congenital vascular anomalies such as Klippel Trenaunay Syndrome, a malformation characterized by improper vascular and lymphatic development and soft tissue hypertrophy [52]. Further investigation should be encouraged in order to examine the interrelated nature of pathological lymphatic and venous development.

### Genetics

A genetic component of chronic venous insufficiency has only recently begun to be identified. Recent areas of study have focused on connexins, alterations in thrombomodulin, matrix Gla protein (MGP), Forkhead box protein C2 (FOXC2), hyperhomocysteinemia, and Type-I plasminogen activator inhibitor polymorphisms.

Connexins comprise a large family of gap junction proteins found to be important in a variety of developmental and physiological processes. Connexin-37 (Cx37) was previously shown to be highly expressed on blood vessel endothelium [53], and recent genetic and molecular analyses have shown that Cx37 is important for proper venous valve development [54]. In their study, Munger et al. demonstrated that venous valves are completely absent in *Cx37*^*-/-*^ mice. Additionally, this group also identified two other connexins (Cx43 and Cx47) that are expressed in venous endothelium in a defined spatial fashion, suggesting that proper localized expression patterns of connexins may be important for proper development of venous vasculature.

Thrombomodulin (TM) downregulates the coagulation cascade by complexing with thrombin and activating Protein C. Le Flem et al. investigated the role of thrombomodulin gene mutations as a potential etiology for venous thromboembolism. Knowing that thrombomodulin can also have a role in proliferation, they also investigated a potential link for thrombomodulin genetic mutations in patients with varicose veins. Three thrombomodulin DNA mutations were evaluated in patients who had participated in the Paris Thrombosis Study, a French case–control study of venous thrombosis. A-1208/-1209 TT deletion was increased to a statistically significant level in patients with varicosities [55]. In addition, this mutation was in linkage disequilibrium with +1418 C/T substitution (Ala 455 Val substitution) in sixth epidermal growth factor-like TM module. This mutation has an effect on the proliferative function of thrombomodulin [55].

The increased genomic expression of several extracellular matrix-remodeling proteins was evaluated in a study by Cario-Toumaniantz et al. Among the genes investigated, increased mRNA expression of MGP was found to be significantly increased in varicose compared with nonvaricose veins. Importantly, the mRNA-to-protein turnover was also investigated, and the authors confirm via Western blotting the elevated levels of MGP in varicose veins. These veins had increased smooth muscle cell proliferation and increased extracellular matrix mineralization, which can be attenuated by warfarin (an MGP inhibitor) and siRNA knockdown of the MGP gene in cultured human saphenous smooth muscle cells [56].

MGP has a role as an inhibitor of matrix calcification. Utilizing gene knockdown techniques, Cario-Toumaniantz et al. were able to demonstrate an increase in mineralization of both varicose and non-varicose smooth muscle cell cultures corresponding to decreased levels of MGP. Microarray analysis also demonstrated alterations in cell organization genes such as actin—which could contribute to the known structural and contractile alterations seen in the varicose vein wall—in addition to an increase in TIMP-1. The shifting balance between TIMPs and MMPs in varicose vein tissues may affect the ability of proteolytic enzymes to degrade components of the extracellular matrix, thereby contributing to the restructuring of the vein wall [56].

The study by Cario-Toumaniantz et al. also demonstrates alteration in gene expression of dermatopontin and tenascin C within the varicose vein but is not replicated in smooth muscle cell culture. Dermatopontin interacts with decorin to regulate collagen fibrillogenesis, while tenascin C induces MMPs and modulates cell-matrix attachment [56].

Mellor et al. illuminated a specific genetic defect in the *FOXC2* gene that they found to be associated with venous valve failure and reflux. *FOXC2* encodes a regulatory transcription factor and is involved in lymphatic and vascular development [57]. The authors investigated the hypothesis that individuals with mutations (frame-shift or deletion) in the *FOXC2* gene present with a greater level of venous reflux in lower limbs. By utilizing venous duplex ultrasound, they found that all 18 patients examined that possessed a *FOXC2* mutation displayed pathological venous reflux. Additionally, patients with lymphatic distichiasis syndrome were also found to have a mutation in this gene with subsequent lymph reflux. Therefore, this study brings to light an important link between genetics and the pathophysiology of primary venous insufficiency, with the suggestion that *FOXC2* is an important gene involved with normal venous and lymphatic valve development [57].

Sam et al., investigated the correlation between hyperhomocysteinemia (HHcy) and chronic venous insufficiency. The investigators found a statistically significant increase in HHcy in patients with venous insufficiency (CEAP 2–6) compared with the accepted prevalence in population (39% vs. 5%). An increase in the severity of venous disease resulted in an increased likelihood of elevated HHcy levels. The most common genetic mutation for HHcy is methylene tetrahydrafolate reductase (MTHFR) C677T polymorphism. Fifteen percent of patients in the study were homozygous for this mutation, compared with 5% in a healthy population [58].

Type I plasminogen activator inhibitor (PAI-1) is a regulator of the fibrinolytic system, and variations in plasma levels of PAI-1 can disrupt the clearance of fibrin. The *PAI-1* 4G/5G gene polymorphism consists of a deletion or addition of a guanosine in the promoter region of the *PAI-1* gene [59]. This polymorphism and its association with chronic venous insufficiency was investigated by Katrancioglu et al. In their examination of 34 patients who present with chronic venous insufficiency, the *PAI-1* 4G allele was found to be increasingly present compared with normal control patients. Additionally, the 4G allele was associated with a 3.25-fold increase in risk for development of chronic venous insufficiency [60].

### Summary

**Figure 2:**
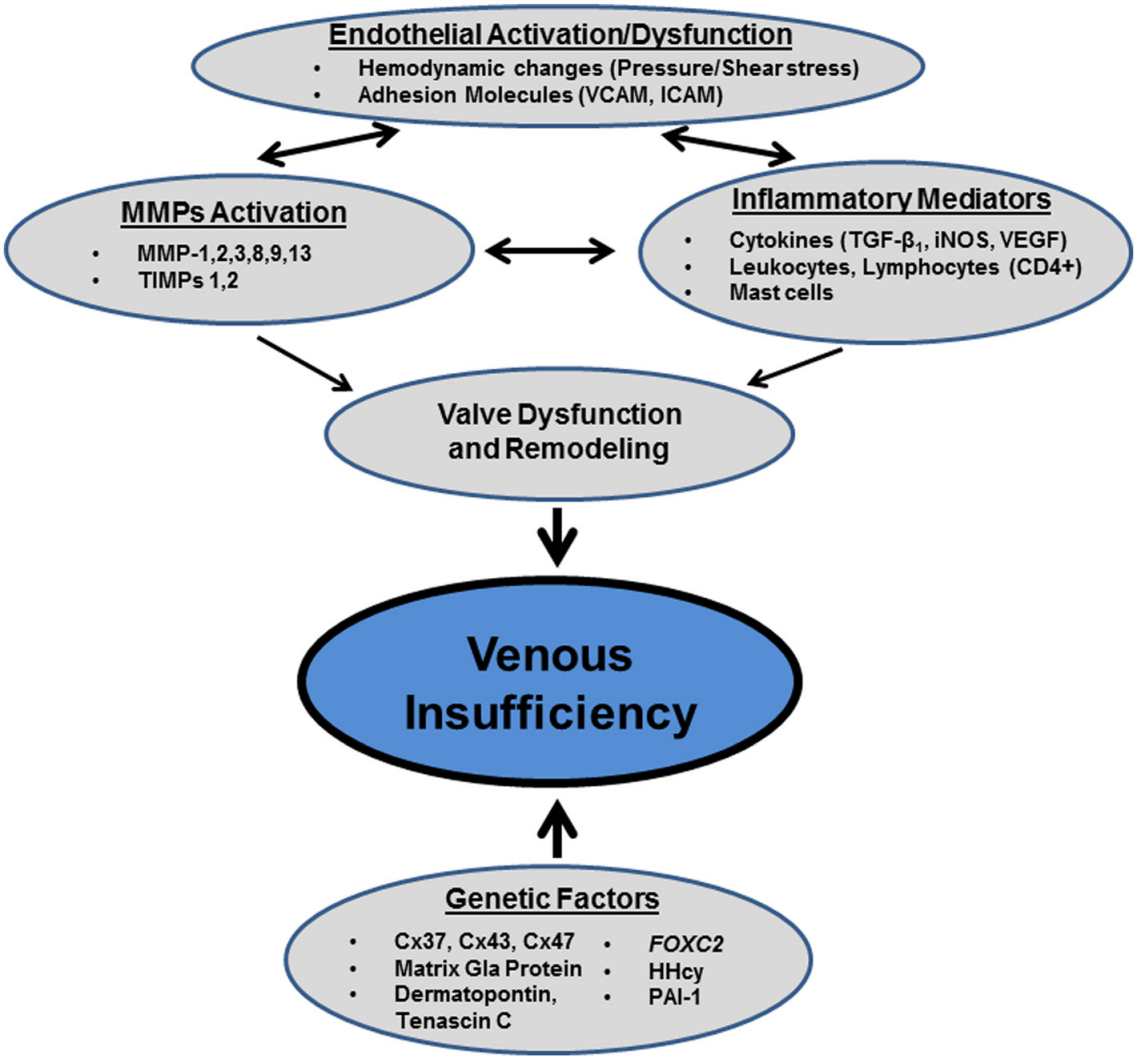
**Schematic representation of the interplay of factors involved in the pathogenesis of venous insufficiency.**

There is increasing evidence to support the notion that among several possible trigger mechanisms, chronic venous insufficiency is to a considerable degree a blood pressure–driven inflammatory disease. Elevated venous pressure and a shift in fluid shear stress generate an abnormal biomechanical environment in venules, in their walls and in valves, which may initiate early activation of degrading enzyme activity and in turn set in motion an inflammatory cascade (Figure 2). A variety of pro- and anti-inflammatory biomarkers can be detected of which no particular one is specific for chronic venous insufficiency. It is possible that a variety of genetic polymorphisms increase the susceptibility to changes mechanical environment and serve as risk factor for chronic venous insufficiency.
